# A Descriptive Methodology for Studying the Ontogeny of Object Play and Breed Differences in Dogs (*Canis lupus familiaris*)

**DOI:** 10.3390/ani13081371

**Published:** 2023-04-17

**Authors:** Karen M. Davis, Adam M. Partin, Gordon M. Burghardt, Cary M. Springer, Julia D. Albright

**Affiliations:** 1 Department of Psychology, SUNY Potsdam, Potsdam, NY 13676, USA; 2 Department of Psychology, University of Tennessee, Knoxville, TN 37996, USA; 3 Department of Ecology & Evolutionary Biology, University of Tennessee, Knoxville, TN 37996, USA; 4 Office of Information Technology, Research Computing Support, University of Tennessee, Knoxville, TN 37996, USA; 5 Department of Small Animal Clinical Sciences, College of Veterinary Medicine, University of Tennessee, Knoxville, TN 37996, USA

**Keywords:** behavioral development, breed differences, domestic dogs, object play, ontogeny, play ethogram, *Canis familiaris*, solitary play, social object play

## Abstract

**Simple Summary:**

The development of object play in animals, including dogs, has been understudied compared to social play behavior. The aims of the present study are to describe the development of a detailed ethogram of object play for dogs that can also be applied to studies of related canids. Moreover, we describe the ontogeny (i.e., the development) of object play in three breeds of domestic dogs from 3–7 weeks of age as they interacted with five different pet toys (objects). Individual behaviors were then categorized into three groups as follows: those that occurred only in the solitary context; those that occurred only in the social context; and those that occurred in both contexts. Theses behavior groups were analyzed for differences across breed and age. Solitary object play developed first, and social object play developed later across breeds. Uncovering early developing breed differences in object play behaviors may aid in understanding both how play develops and the role that selection and domestication has played in the evolution of dogs and their behavioral variability.

**Abstract:**

Play behavior is a prominent aspect of juvenile behavior for many animals, yet early development, especially play with objects, has received little attention. Our previous study on object play introduced our general methods, focusing on litter differences in the developmental trajectory of object play and toy preferences. Here, we present a detailed ethogram of more than 30 observed object play behaviors. We focus on breed differences in the development of play in the three following breeds: Welsh Terriers, Vizslas, and standard Poodles. Puppies were video recorded from 3 to 7 weeks of age at half-week intervals upon the introduction of a standard set of five toys into their home environments. Ten minutes of video from each session for each puppy were analyzed using the Noldus Observer XT program. Aside from analyzing individual behaviors, they were also grouped into three behavioral categories. These were behaviors that occurred only in a solitary context, only in a social context, or in both contexts. Solitary object play developed first, and social object play developed later across breeds. There was a significant three-way interaction between breed, developmental age, and the context in which play occurred. Pairwise comparisons within each breed, age, and context are discussed, but a prominent result is that the onset of many behaviors occurred later in Welsh Terriers compared to the other breeds.

## 1. Introduction

Play is a heterogenous set of behaviors observed across many contexts and ages, in both vertebrate and invertebrate species [1]. However, the functions, mechanisms, and evolution of play remain elusive. Many hypotheses have been advanced, and no single function of play is likely to be applicable given its diversity [2,3]. Play type also differs greatly and has historically been characterized as social, object, or locomotor/rotational [2]. Object play has been studied extensively in adult animals [4]. The association of object play with environmental exploration [5] has been noted for many years. Indeed, play with an object usually follows simple exploration of the object, prompting some to postulate that play is a complex version of exploration [6,7,8] or a form of self-induced sensory stimulation [9,10]. More recent hypotheses suggest an integration of play into pre-existing exploratory mechanisms, and this may function by helping an animal assess novel objects and overcome neophobia where appropriate [2,11,12,13]. Not surprisingly, object-directed play is the most ubiquitous category of play reported across all species [2,14].

The domestic dog (*Canis lupus familiaris*) provides an excellent model for the study of play due to the prevalence of play throughout much of a dog’s life, as well as the diversity of social, object, and locomotor/rotational play that they perform. Neoteny, a type of paedomorphism [2], whereby some juvenile (wolf) traits are retained into adulthood during the domestication process, has enhanced playful behavior in adulthood for dogs [15,16,17], although play is seen in adults of many wild species, including wolves; thus, it is not just an artifact of domestication [18]. Dogs display socially playful conspecific and interspecific behavior; play with objects, including many commercial dog toys; and display movements including intense bursts of running, jumping, or spinning without obvious purpose (locomotor/rotational play). Overlap of categories is possible, e.g., dogs often incorporate objects and intense locomotor patterns into social play (retrieving, keep-away, tug-of-war, etc.). The majority of canine play research has focused on social play [19,20]. However, a recent study of social and solitary (including object) play compared adult dogs from several breeds that differed in the types of predatory behavior they retained (retrievers and herders vs. livestock guarding). Solitary play levels were higher across all contexts than social play, but environmental context influenced solitary play [21]. While they found no differences in social play levels across breeds or context, they did find breed differences in solitary play, with retrievers engaging in it more overall than livestock-guarding dogs.

The ontogeny of object play has been described more extensively in another domestic species, the cat, for which the behavior closely resembles predatory and prey-handling behavior. Hunger and object play may be directly proportional in kittens [22]. These pieces of evidence have prompted some support for a shared hunger and hunting motivational system, in which object play is simply unrefined predation [4,23]. However, others point out that object play is very common in experienced adult cats and some obvious motor pattern differences from the full predatory sequence are present (e.g., rapid approach/retreat and lack of object ingestion) [24], indicating the cat is capable of recognizing the difference between prey and play items [2,25]. As in most species, including domestic dogs, play is more frequent in healthy individuals not under undue stress [2].

Playful object interactions in adult dogs differ depending on context. In solitary object play, predatory-like motor patterns are prevalent and most intensely triggered by “prey-like” aspects of the object (toy), such as erratic movements and high-pitched noises [26]. Studies [27,28] have also documented that soft items are preferable to hard toys in dogs and wolves. Solitary object play is very susceptible to habituation, or lack of interest, after some period of time [29]. Object play in adult dogs is often seen in a social context, and objects are used differently depending on the play partner. Adult dogs seem to use objects to maintain playful interactions with a human, but the same dogs show more competition when the same toy is used in dog–dog play [30,31,32].

Examining the ontogeny of a behavior through a comparative lens can provide insight into its origins [33]. Mapping differences onto phylogenies and incorporating the species’ natural histories may also provide a window into the function of a behavior [34]. Evidence suggests object play in wild canid pups may reflect adult hunting strategies. For example, object play contained little competition in bush dogs, a species that hunts large prey in packs, compared to the crab-eating fox, a semi-social species that hunts individually [35]. Given the morphological and behavioral diversity of the domestic dog, it is not surprising that play behaviors have been shown to differ by breed [21]. Research from our earlier work [27] and previous findings from others [36,37,38,39,40,41,42] indicate that not only species [35,41,42] but also breed effects on social and object play can be detected prior to 7 weeks of age.

The primary developmental stages in domestic dogs have been described by Scott and Fuller [43] from comparative studies on the development of five dog breeds, namely Basenjis, Beagles, Cocker Spaniels, Shelties and Fox Terriers, raised in a kennel environment. The developmental periods described by Scott and Fuller are still used today and are labelled the neonatal period (birth to eyes completely open, about 12–14 days), the transitional period (eyes open to startle response, ~12–14 days or 20–25 days, i.e., 2–3 weeks), the socialization period (3 weeks or 20–25 days to ~12 weeks), and the juvenile period (~12 weeks to sexual maturity, i.e., 6 months to a year). Scott and Fuller noted both individual variation in pups and across breeds in developmental milestones. During the neonatal stage pups are blind and deaf, with poor thermoregulation and mainly respond to nursing, the scent of their mother, and temperature. The transitional period includes the opening of the eyes with pups starting to see light and dark, the startle reflex at the start of hearing, and the start of motor development with crawling and the start of walking. The socialization period includes the rapid developments of social behavior patterns and relationships with litter mates. The start of teeth eruptions occurs at 20 days and continues through weaning. Weaning is gradually started by the mother at 5 weeks of age and final weaning occurs between 7–10 weeks of age. The juvenile period (~12 weeks to sexual maturity, which can range from 6 months to 1 year in dogs) of rapid growth ends at 16 weeks when pups are 2/3 adult size and have adult teeth. The juvenile stage is marked by increases in motor strength and skill rather than development of new behaviors. These same developmental periods are used for wolves because their overall development is similar [44]. However, wolf pup motor development is 2 weeks ahead of dogs [45]. Startle responses occur earlier in wolves (16–17 days) [46], but maturity does not occur until 22 months in wolves [44].

Comparative studies of the development of play, particularly object play, are needed to explore potential functions of play and how environmental and social grouping might affect its developmental onset and characteristics [35]. Few studies have looked at the early development of object play during the socialization periods [27,28,35,42,43] in canids when behavior patterns are first emerging, and differences in emergence and repertoire may give us clues of adult play function.

Our previous study in dogs [27] examined the development of object play from 3–7 weeks of age, focusing on time spent playing with objects, toy preferences, and the development of play complexity. The current study builds on this preliminary study, including more detail with more litters and focusing on the ethogram, breed differences, social contexts, and individual behavioral development. As part of a larger project gathering data on social and object play in many breeds of dogs [27] and wolves (*Canis lupus*) [28], the aim of the present study is to evaluate the ontogeny of object play in three breeds of domestic dogs as they interacted with five different pet toys (objects). The specific objectives included the following: (1) provide descriptive details underlying the ethogram presented in previous [27] and present studies [28]; (2) apply the ethogram to behaviors observed as the puppies interacted with the objects and group these individual behaviors into larger play contexts; (3) assess the effects of age (3-7 weeks of age) on the duration of play behavior both in larger play contexts and individual behaviors within contexts; and (4) examine the behavioral development of object play across three breeds of dog with diverse histories. Uncovering breed differences during ontogeny in the type and frequency of object play behaviors may aid in understanding both how play develops and the role that selection and domestication has played in the evolution of dogs and their behavioral variability.

## 2. Materials and Methods

### 2.1. Subjects

The subjects were 7 litters (L) of puppies, totaling 42 animals (18 males, 24 females) from 3 breeds of dog (*Canis lupus familiaris*). The breeds were Welsh Terriers (3 litters, 11 puppies; L1: 1M, 2F; L2: 2M, 4F; L3: 1M, 1F), standard Poodles (2 litters, 17 puppies; L1: 4M, 6F; L2: 3M, 4F), and Vizslas (2 litters, 14 puppies; L1: 3M, 4F; L2: 4M, 3F). These three breeds were chosen as part of larger study designed to compare dogs from different phylogenetic clades [47] and American Kennel Club groups “www.akc.org (accessed on 23 February 2023)” with a long-term goal of studying the influence of phylogeny and function (herding, retrieving, guarding, etc.) on the development of behavior. The subjects are identical to those in the earlier study [27], other than the addition of Welsh Terrier L3, which was added to create more equivalent breed sample sizes. To minimize kennel effects, different breeders with similar types of within-home housing, medical care, environmental enrichment, and husbandry practices were chosen for this study. Participating breeders provided puppies with a similarly high level of sensory and social stimulation (i.e., a variety of objects, sounds, indoor and outdoor locations, substrates, and human handling). All litters were bred from different dams and sires to reduce potential parental genetic or maternal effects on overall breed results. Furthermore, all breeders were in different geographic locations with the exception of the L1 and L3 Welsh Terriers, which were born within a few weeks of each other to different dams and sires but housed in separate kennels in the same location.

As this was part of a larger study, whenever possible, video recordings of the litters began when the pups were 14 days old (2 weeks). For some litters (L1 Poodles, and L1 and L2 Welsh Terriers), we were also able to observe and record the pups for more than 7 weeks (up to 9–11 weeks). Therefore, our pilot data used to create the ethogram and observer coding scheme listed below were enriched by this larger dataset. Researchers’ classification of pup weeks varies [45,48]. Our meaning of week usage here is the same week scheme used in [27,28] and is clarified here by denoting the equivalent day age of each week timepoint as follows: 3.0 weeks (21 days); 4.0 weeks (28 days); 5.0 weeks (35 days); 6.0 weeks (42 days); and 7.0 weeks (49 days). Mid-week sessions were made on day 3 or 4 of each week.

### 2.2. Stimuli

**Figure 1:**
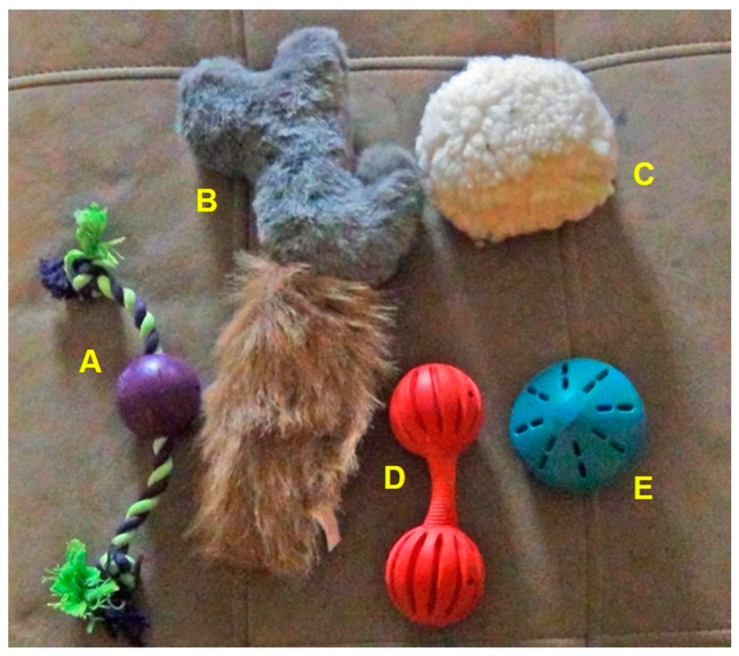
The 5 different PetSafe research toys (objects). These objects are labelled as seen in the figure: (**A**) a braided cloth rope attached to hard rubber ball; (**B**) a squirrel; (**C**) a white plush puff; (**D**) red rubber bar-bell-shaped bone; (**E**) and a blue hard rubber disk.

Each litter was tested with their own standardized set of 5 different PetSafe^®^ toys (hereafter refer to as “objects”) as follows: plush squirrel (Wild Squirrel^TM^); plush puff (Pogo Plush^TM^); braided cloth rope attached to a hard rubber ball (Roly Rope^TM^); a blue hard rubber disk (Twist ’n Treat^TM^); and a red rigid rubber bar-bell-shaped bone (Waggle^TM^) (See Figure 1). The objects were available in one, two, or three different sizes depending on the toy type. Each litter was assigned one set of toys based on breed for the duration of filming. Details regarding object sizes and provision to puppies based on breed are provided in the earlier study [27].

### 2.3. Procedure

The testing areas and recording methods were identical to those described previously [27]. Briefly, 90 min video recordings were systematically collected twice a week (at full and half-week age timepoints). Although some pups were video recorded from 14 days old (2 weeks), data presented in this paper start at 3.0 weeks as not all litters were recorded at the week 2 timepoint. Video available for the earlier weeks showed that puppies rarely approached or interacted with the objects before 3 weeks of age, irrespective of breed. Data analysis was terminated at 7.0 weeks because many pups departed the breeder’s facility at this age. Mid-week sessions were made on day 3 or 4 of each week.

Puppies were individually identified for video recording with either removable colored paper collars or similar collars provided by the breeder. If the breeder did not want the puppies to always wear collars, collars were placed on the puppies 10 min before the start of the 90 min of the video-recorded sessions. Each litter was provisioned with a new set of the 5 standardized toy objects as noted above. The same set of objects was used throughout the rest of filming unless an object was lost or destroyed, and then a replacement object was supplied. All non-study objects and the dam were removed from the enclosure area 10 min prior to the video-recording session. Video recording occurred when standardized study objects were introduced to start the session, which were removed after each filming session to help ensure that long-term habituation was minimal. If individuals in a particular litter displayed fear of any object during the first session, we allowed the owners to leave the objects with the pups for part of the following day until the pups were no longer avoiding the objects. All research objects were present for all video sessions, with the exception of 1 object (plush squirrel), which was missing during one session with the L1 Poodles. The object was subsequently replaced during the next video-recording session.

Puppy behavior was recorded using digital Sony camcorders (Sony HDR-CX405) for all litters, except for the litter of 6 Welsh Terriers, for which the owners used their own digital camera. Puppies were contained in areas that increased in size proportional to litter age and breed size so that all pups were visible on screen for almost the entire time while giving them ample room to move and play. All litters started out housed indoors inside whelping boxes (approximately 122 × 122 cm) at 2 or 3 weeks of age. Housing increased in size up to about 4 m^2^ as the puppies grew in size and included both indoor and outdoor areas. All litters were filmed between 1000 and 2130 h during times when breeders found the puppies were most active and the owners were available. Prior to weaning, pups do not have a strong circadian rhythm and are not awake and active for much longer than 10 min at a time. We continued the same 10 min continuous play period as the dogs aged.

### 2.4. Behavioral Analysis

A focal observation method was used to assess the first 10 min (from the pre-recorded larger 90 min video) after the introduction of the standardized objects was analyzed for each puppy or, if the animals were sleeping at the start of object introduction, the first 10 min after the puppies awakened. We focused on the first 10 min as pilot data demonstrated pups generally showed less interest in the objects later in the filming period. This approach is supported by previous research in adult kennel-raised dogs, suggesting habituation to objects [29]. Week and mid-week sessions were coded for each pup, totaling 20 min per week of coded video for each pup.

Detailed systematic data collection, coding, and analysis of dog behavioral repertories, counts, and sequences were carried out using the Noldus Observer XT (version 13), which synchronized videos with the Observer program and behavior coding template so that multiple passes could be accurately carried out for each individual pup. In this way, using the behavior coding template, observers were able to separately analyze the same 10 min videos with continuous all-occurrence focal animal sampling [49] of play bouts for each individual pup with each instance and sequence of behaviors synchronized in chronological order.

Videos were coded by noting the individual puppy, object, and behavior displayed in relation to the object. If an interval of greater than 2 s elapsed between behaviors, they were coded as separate instances. Five trained coders (AMS, GSS, TA, TV, and CW) unfamiliar with any hypotheses or predictions performed most of the Observer XT scoring. Observers were trained in the ethogram prior for coding or collecting IOR. Once observers were trained in the ethogram, we combined mouth/chew. We focused on behaviors that occurred during object play (Table 1) and not inapplicable times, such as when the pups were not interacting with the toys or were focused on social play. We also focused on the functional behaviors such as *paw* without modifiers, i.e., *smack* or *touch*, to decrease interobserver error and because coded data used for this study did not include the modifiers separately. Once all observers agreed on behaviors and how to use the ethogram, we started coding and, at this time, took IOR for the sequence of behaviors coded to further verify the ethogram and interobserver reliability for behaviors and sequences of behaviors within each play bout.

**Table 1:** Object Play Ethogram. Behaviors exhibited during interactions with objects. Each behavior is followed by noting the context in which the behavior in the ethogram occurs: only one animal engages in the behavior (solitary/SOL); two or more animals engage in the behavior (social/SOC); behavior occurs in both solitary and social contexts (both/BOTH). In two cases, the name used changed between this paper and [27] The former name is noted under the definition.

Behavior	Definitions
Agonistic Behavior	Behaviors seen: snapping; biting; piloerection; agonistic pucker; growling; or rushing another puppy (see Table 2 for more detail).
Solitary Behaviors (SOL)	
Bite (SOL)	Closing jaw with mouth or teeth (pups can exhibit this behavior before they have teeth) on an object and then quickly releasing.
Carry (SOL)	Picking up and holding an object with teeth and moving greater than 2 steps before depositing the object; the object does not touch the substrate in transit (compared to drag).
Chew (SOL)	Repeatedly manipulating an object with mouth or teeth. Using teeth may include biting (usually with incisors and canine teeth) or gnawing (using the molar teeth) the object; may involve ingesting pieces of the object.
Dig (SOL)	Rapid extension and flexion of alternating forelimbs on an object or pen substrate adjacent to the object.
Drag (SOL)	Picking up an object with mouth and moving greater than 2 steps away before depositing the object; portion of the object remains in contact with substrate in transit.
Grab (SOL)	Closing mouth or teeth on an object for at least 2 s while remaining stationary or moving no more than 1 full step.
Hold Object (SOL)	Possessing an object either in mouth or between forepaws while stationary.
Lie on Object (SOL)	Placing body over an object; usually pup rolls on top of the object.
Lick (SOL)	Extruding tongue from mouth and passing over an object.
Nose (SOL)	Touching an object with nose. Modifiers: *Nudge*: moving an object with nose. *Touch/sniff*: touching nose to an object without resulting in movement of the object (may include sniffing the object).
Pickup and Drop (SOL)	Repeatedly picking up and immediately dropping object.
Tear (SOL)	Grabbing an object with teeth and pulling it with force; usually the object is held down by forepaws as teeth and head pulls up.
Toss (SOL)	Flexing neck down and then rapidly extending neck while releasing the object held in mouth.
Tug-pull (SOL)	Grabbing with teeth and pulling back on a non-movable object or another puppy that does not tug in return; often accompanied by growling. The other puppy may let go immediately or hold onto the object for a few seconds before releasing it but does not tug back. (Previously called pull-tug [27].)
Behaviors occurring in both contexts (BOTH)	
Approach (BOTH)	Moving from one area in the enclosure towards an object or puppy in another area intentionally, i.e., the pup is clearly headed toward the object or pup and does not merely run into the object or pup.
Approach-retreat (BOTH)	Repeatedly stepping or rocking toward and then away from the object or puppy with an object.
Avoid (BOTH)	Moving body or head away from object or another puppy; may be as subtle as looking away or turning the head away, or may include turning body and crawling, walking, or trotting away from the object and/or pup.
Exaggerated Approach (BOTH)	Moving toward an object, puppy with an object, or approaching another puppy while carrying an object with a bouncy gait at a speed greater than a walk, often with side-to-side motion movement of head and shoulders that is exaggerated from normal approach.
Grab-headshake (BOTH)	Grabbing an object with mouth or teeth, followed by rapid side-to-side movement of head with an object in mouth. This often includes growling. Pups less than 5 weeks display uncoordinated head and body shake. At approximately 5 weeks, the movement becomes more limited to the head and varies in speed and intensity, often seen during tug-of-war. Other names used for this behavior in adult wolves include bite shake [50] *and headshake* [51], and it is labelled *bite-shake* in our previous dog paper [27].
Paw (BOTH)	Extending forepaw toward an object with or without making contact with the object. Modifiers: *No-contact*: extending paw toward the object without touching it. *Smack*: extending and pressing down on the object with paw. *Touch*: extending paw and lightly touching the object with paw. *Bring-in*: Extending and flexing paw on the object to rake the object toward body.
Play Bow (BOTH)	Dropping elbows to substrate with hind limbs remaining in an upright position; may include side-to-side movement.
Pounce (BOTH)	Rapidly jumping towards another pup or an object with only front limbs leaving the ground.
Stand Over (BOTH)	Standing over an object or another puppy lying down with an object.
Social Behaviors (SOC)	
Chase (SOC)	Moving faster than walk in pursuit of another puppy that is in possession of an object.
Guard (SOC)	Standing over an object when approached by another puppy; may be accompanied by lunging at the other puppy, piloerection, or other agonistic behaviours.
Keep Away (SOC)	Carrying an object and turning head or body away from an approaching pup; gait is usually faster than a walk and is followed by chase from the other pup.
Leap (SOC)	Jumping with all four feet off the ground towards another puppy with an object.
Paw Face (SOC)	To extend or wave the paw to touch another puppy in the face that has an object. Modifier: *Paw no-contact/paw touch/paw smack* (see above)
Tackle (SOC)	Jumping on or running into another pup with an object, usually knocking other pup over; may occur during a keep-away/chase episode.
Tug-of-war (SOC)	Two or more pups grasping an object in mouth and pulling back in opposite directions; weight is shifted to back limbs; may growl, grab-headshake, or paw at the other pup; bout ends with at least one pup dropping the object.

**Table 2:** Observed behaviors not included in play data coding or analysis. (A) Vocalizations observed in pups from 3–7 weeks of age. (B) Agonistic behaviors. Behaviors exhibited during social interactions that did not include objects. These definitions are adapted from already existing definitions in [51].

Behavior	Definition
A. Vocalizations	
Bark	Short, loud, and relatively high-pitched vocalization with abrupt onset; frequency modulation often has both tonality and noise and is subject to rapid repetition.
Growl	A throaty, low-frequency rumbling.
Whimper	Emitting repeated (approximately 2–3 times per second) high-pitched monotone vocalization on exhalation.
Whine	Emitting repeated (usually longer than 0.5 s) vocalization falling pitch.
B. Agonistic Behaviors	
Agonistic Pucker	Vertically retracting the lips with either the corners of the mouth forward, showing canines and incisors only (offensive) or corners of mouth drawn back exposing pre-molars (defensive).
Growl	A throaty rumbling vocalization that is usually low pitched and can be used defensively or offensively in aggressive interactions.
Hackles	Piloerection of the fur along the spine. Hackles can be scruff/withers (H1), back (H2), rump (H3), and tail (H4). Piloerection increased with arousal usually in sequential order ranging from H1 to all 4 hackles (H1234) depending on arousal level. Nonsequential combination of hackles also occurred such as H13.
Lunge	A direct rapid approach at another puppy that is close to the object, usually with an agonistic pucker, snap, and sometimes a bite. The movement may take the form of a jump or a few running steps.
Snap	A rapid bite. When the jaws come together, the teeth make an audible sound. This is often an air snap where the pup does not make contact with the other pup but could include contact with the other pup.
Rush	A short run directed at another puppy.
Yelp	A quick sharp shrill bark or cry.

Strong interobserver reliability for coding the videos was established looking at 10% of the videos across breeds. Cohen’s kappa averaged across all observer pairs for all breed observations was 0.94 for total play duration. Cohen’s kappa for duration sequence averaged across all pairs for all breed observations was 0.96. Both duration and duration sequence were calculated in the Observer program. Cohen’s kappa was calculated by hand for agreement of behavior exhibited, sequence of behaviors, and number of bouts combined, as this was how the continuous video was coded. This Cohen’s kappa was 0.80, averaged across all observer pairs for all breed observations. See Appendix A Table A1 for the average kappa of each observer pair for behaviors, duration, and duration sequence. There was 100% reliability in terms of the identification of individual pups. Statistical analysis of the data was carried out using SAS and SPSS 22 statistical software.

### 2.5. Ethogram Development

We created an ethogram of all object-related behaviors by adapting elements found in earlier canid play ethograms and using a sample of our dataset (see Table 1). Play initiation behaviors in our ethogram, including *exaggerated approach*, *approach-retreat*, and *play bow*, originated from research on the comparative social play development of wolves (*Canis lupus*), coyotes (*C. latrans*), and Beagles (*C. lupus familiaris*) during the socialization period [41]. We also included the terms *pick-up* and *drop* from non-domestic South American canid observations [35]. Vocalization definitions were derived from adult dogs [52] (*bark*) and wolves [51] (*growl*, *whimper*, *whine*, and *yelp*). However, very few studies of either object play or puppies prior to 8 weeks of age have been conducted, and most behaviors used in this study were created de novo based on our observations [27]. Ethogram (Table 1) creation came from extensive examination of the videos of a variety dog breeds at each age collected during the pilot phase of this project. Table 1 represents the final ethogram for all object-related behaviors used to code the videos and collect frequencies and counts for each behavior. During pilot observations and observer testing of the ethogram prior to coding, *mouth* and *chew* were combined, as noted in the ethogram (Table 1), and were coded as *chew* during data coding due to the difficulty in distinguishing the subtle differences from one camera angle. Table 2A includes agonistic behaviors initially observed in a social object context that then often quickly transitioned to non-object social agonistic behaviors. Table 2B includes all observed vocalizations. The behaviors in Table 2 are based on our observations and are adapted mostly from wolves [51]. The behaviors and vocalizations listed in Table 2 were not used to code data. All data coding occurred using Table 1.

This study focused on the behaviors the pups displayed with the objects, either by themselves or with other pups, but not human–pup interactions. Therefore, we did not include any play with objects that included a human or any time period when a human was present in the pup enclosure as they were often a distraction. Because the video was continuous, if human interactions occurred during a sample period, we marked it as a “human interaction” category and excluded them from the data coding and sample period. The instances of human interaction were very rare and short in duration, comprising only 0.13 percent of the overall duration, and did not warrant adding extra overall duration to the sample periods.

### 2.6. Object Play Contexts

The individual behaviors in the ethogram were further categorized into three object play contexts (referred to as the play context) as follows: play behaviors performed only by a solitary animal (solitary/SOL); play behaviors performed only by two or more individuals (social/SOC); and those that were observed in both solitary and multiple individual contexts (both/BOTH). The assigned play context is indicated for each individual behavior in the ethogram (Table 1). The total observed counts and duration of individual behaviors were summed for each play context by breed standardized across a 10 min time frame for each week and half week in Table 3 for descriptive purposes. For statistical analysis, mean durations were used throughout.

#### Statistical Analysis

Beginning of the week and mid-week behavior durations were averaged to represent the full week for statistical analysis. Weeks 3, 4, 5, 6, and 7 were compared. Repeated-measures linear-mixed-model ANOVA, using a Kronecker product unstructured compound-covariance structure with random effect for litter, was used to test the between the subject effect of breed and the within-subject effects of age and play context. Due to the non-normal distribution of residuals, the analyses were run on ranked transformed behavior durations. All model assumptions were met.

Significant three-way interactions were explored by running additional repeated-measures linear-mixed-model ANOVA for each play context, testing the effects of breed and age within each context at each week age. Significant breed effects for each play context were tested using pairwise comparisons. Significant week-of-age differences for play context were tested using sequential contrasts, comparing each week to the subsequent week. Individual behaviors of play context that had significant breed differences were analyzed in the same manner of the model used and post-hoc tests. Analyses were run using SAS software version 9.4. (SAS Institute, Inc., Cary, NC, USA) with an alpha of 0.05.

Statistical analyses were performed on rank-transformed data. The resulting graphs represent the mean ranks, although means with standard deviations for play context data are found in the graphs, as well as in Appendix A Table A2, Table A3 and Table A4.

We are aware that individuals within a litter reared together are not independent. However, social interactions in relation to the objects are a critical component of play and testing the littermates together was essential to properly evaluate our study objectives. We also felt that testing individuals in isolation would have created undue distress on young puppies. Therefore, we controlled for litter by making it a random factor in the model and focused on breed differences for this specific study.

## 3. Results

### 3.1. Behavioral Repertoire

#### 3.1.1. Basic Ethogram

The ethogram includes a total of 31 object-related behaviors classified into the three play contexts (Table 1). Seven agonistic behaviors were observed, and four vocalizations are listed in a separate agonistic behaviors and vocalizations table (Table 2).

#### 3.1.2. Behavior Observations

Table 3 shows the total counts and durations of all primary ethogram behaviors for each breed collapsed across the total observation period (3–7 weeks). To examine the changes in behavior over time, the total number of pups exhibiting each behavior at least once per time point (week and mid-week) collapsed across breed is shown in Table 4. Appendix A Table A5 shows the total counts and duration for each time point collapsed across breed. Simple investigative behaviors, such as *chew* and *nose*, were the most prevalent, whereas *grab*, *paw*, and *carry* occurred with moderate frequency. *Nose*, *chew*, or *paw* were the first object-directed behaviors to develop. The total counts of each behavior generally increased over time, although some plateaued or decreased. *Guard* was not observed in Poodles, Terriers, or Vizslas at 3–7 weeks of age.

#### 3.1.3. Behaviors Excluded or Combined for Statistical Analysis

A few behaviors were combined for the statistical model. After all behaviors were coded using the ethogram (Table 1) from the video, some behaviors were excluded or combined with similar behaviors for statistical analysis as they could not be individually included in the model due to low occurrence. *Approach-retreat* was collapsed into *exaggerated approach* because both behaviors were appetitive play behaviors—either an invitation to play with another puppy or an intent to play when directed at an object.

A number of behaviors were excluded from further analysis after descriptive statistics were presented (Table 3) due to low occurrence. Each of the following behaviors comprised under 13 total counts across all breeds for the total observation period (3–7 weeks) and many were only observed in 1 breed, thus they were removed from further analysis: *agonistic*, *avoid*, *dig*, *leap*, *lie on object*, *stand over*, *toss*, and *tackle*. Only object-related behaviors that could be clearly discerned as play were evaluated. Agonistic behaviors were not included in the statistical analysis (see the Agonistic Behavior Section below) and were removed from further analysis after descriptive statistics were presented (Table 3).

##### Agonistic Behavior

A separate ethogram table was created for agonistic behaviors, such as *growl*, *snap*, and *bite* (Table 2). Agnostic behaviors associated with the objects were rare in puppies at this age, observed in only three instances in the Vizslas (Table 3). Therefore, although agnostic behaviors as noted in Table 1 were coded according to object interaction, they were eliminated from statistical analysis. Agonistic behaviors often alternated or occurred simultaneously with other social behaviors, such as wrestling or *tug-of-war*. While rough-and-tumble play and agonistic play fighting are well-known categories of play [3,12], the ontogeny of these behaviors deserves further investigation. It was beyond the scope of this study to determine the motivational state of the puppies when they displayed agonistic types of behaviors. Behaviors in Table 2 were excluded from further analysis here.

#### 3.1.4. Ontogenetic Changes in Play Behaviors

The appearance of some behaviors altered as the puppies’ neuromuscular systems developed. For example, early instances of *grab-headshake* involved strong motion of the puppy’s entire body, whereas the action became centered on the head with little body movement as motor control advanced. The rate of head shaking also increased with maturity. When pups first engaged in *tug-of-war* behavior, they tended to position their bodies in a more parallel arrangement, and it was not until later weeks that puppies moved to the opposite ends of the toy to tug against one another. The duration and intensity of *tug-of-war* and *tug-pull* (solitary version of *tug-of-war*) also increased with age.

### 3.2. Play Context

Play context refers to the social setting in which the behaviors in Table 1 (excluding vocalizations) were observed. Of the 31 object behaviors (Table 1) after exclusions and term combining, 21 were included in the statistical analysis of play contexts (see Appendix A Table A2, Table A3 and Table A4), 12 in the solitary only context (SOL), 4 in social only context (SOC), and 5 in both social and solitary contexts (BOTH).

#### 3.2.1. Overall Interaction

**Figure 2:**
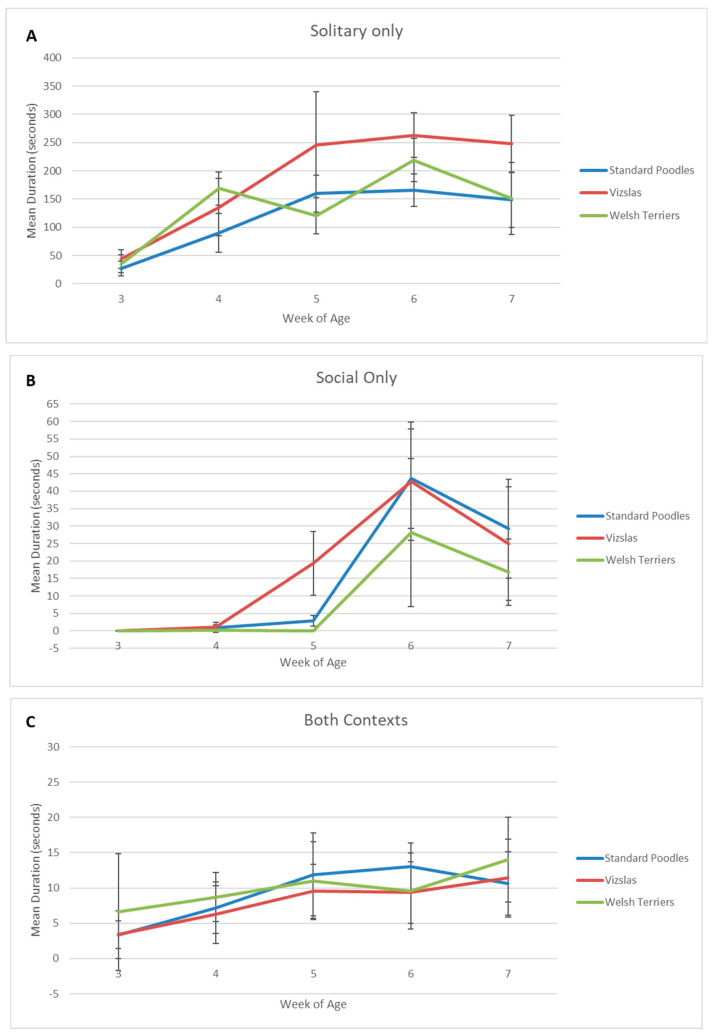
Play Context. Mean durations in seconds for individual pups across breeds for each play context: play behaviors performed (**A**) only by a solitary animal (solitary/SOL); (**B**) only by two or more individuals (social/SOC); and (**C**) those that were observed in either solitary or multiple individual contexts (both/BOTH) by age in weeks 3–7 with 95% CI.

There was a significant three-way interaction between breed, age, and play context [F(16,259) = 2.45, *p* = 0.002]. To explore this three-way interaction, mixed-model ANOVA was used to test for breed by age interaction within each play context. Within each play context, significant interactions were further examined by comparing breeds within each age. Additionally, age was tested sequentially within each breed (see Figure 2). Mean durations are reported for SOL, SOC, and BOTH in Table 5 from untransformed data, although stated statistical differences are based on rank-transformed data.

#### 3.2.2. Behavior Development by Breed

##### Solitary

For SOL behaviors, the interaction of breed and age (measured in weeks so age is labeled in weeks from here on) was not significant, indicating that SOL behaviors did not change differently over time for any breed [F(8154) = 1.26, *p* = 0.27] (Figure 2A). There was a significant main effect of week [F(4154) = 31.65, *p* < 0.001] but no main effect of breed [F(2,4) = 1.15, *p* = 0.41].

Across breeds, SOL behaviors had a significant increase between weeks 3 and 4 (*p* < 0.001), when SOL behavior onset occurred, and weeks 5 to 6 (*p* = 0.028). SOL behaviors significantly decreased between weeks 6 and 7 (*p* = 0.024) (Figure 2A). There was no significant change between weeks 4 and 5 (*p* = 0.27).

##### Social

For SOC behaviors, there was a significant interaction between breed and week [F(8154) = 3.97, *p* < 0.001]. When comparing breeds within each week, breeds did not differ during weeks 3 (*p* = 0.95), 4 (*p* = 0.72) as social object play was rare for all three breeds for the first 2 weeks (see Table 5, Figure 2B); however, they differed in week 5 (*p* = 0.007). In week 5, Vizslas exhibited more SOC behavior than Terriers (*p* < 0.001) and Poodles (*p* = 0.04), while Poodles did not differ from Welsh Terriers (*p* = 0.15) (Figure 2B). In weeks 6 (*p* = 0.07) and 7 (*p* = 0.87), there were no differences between breeds, indicating that the Terriers and Poodles caught up to the Vizslas by the end of our observation period.

When comparing week age within each breed, SOC behaviors for Poodles did not change from week 3 to week 4 (*p* = 0.18) or between 4 and 5 (*p* = 0.10), but they did significantly increase at week 6 (*p* < 0.001) and significantly decrease at week 7 (*p* = 0.003) compared to the previous weeks (Figure 2B). Vizslas’ SOC behavior duration also remained steady between weeks 3 and 4 (*p* = 0.45). They significantly increased at week 5 (*p* < 0.001) and week 6 (*p* = 0.003), then decreased at week 7 (*p* = 0.002) (Figure 2B). Terriers’ SOC behavior only significantly increased between weeks 5 and 6 (*p* < 0.001). Duration did not change between weeks 3 and 4 (*p* = 0.82), 4 and 5 (*p* = 0.82), or 6 and 7 (*p* = 0.96) (Figure 2B).

##### Both

For BOTH behaviors, interaction of breed and week was not significant, indicating that BOTH behaviors did not change differently over time for any breeds [F(8155) = 0.70, *p* = 0.69] (Figure 2C). There was a significant main effect of week [F(4155) = 10.32, *p* < 0.001] but no main effect of breed [F(2,4) = 0.05, *p* = 0.95].

Across breeds, BOTH behaviors had a significant increase between weeks 3 and 4 (*p* < 0.001) when BOTH behavior onset occurred (Figure 2C). There was no significant change between weeks 4 and 5 (*p* = 0.11), 5 and 6 (*p* = 0.92), or 6 and 7 (*p* = 0.95).

#### 3.2.3. Individual Behaviors within Each Group

Means and standard deviations for individual behaviors for each play context are reported in Appendix A Table A2, Table A3 and Table A4. Because SOC was the only play context that significantly differed by breed, individual behaviors in this group were further analyzed. There was a significant breed by week interaction for SOC individual behaviors [F(24,741) = 3.52, *p* < 0.001].

**Figure 3:**
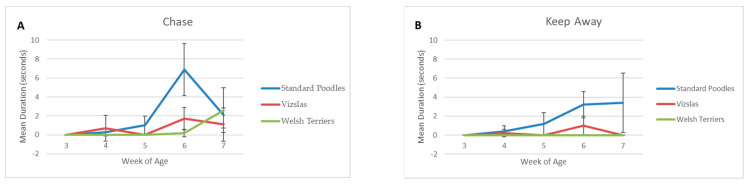

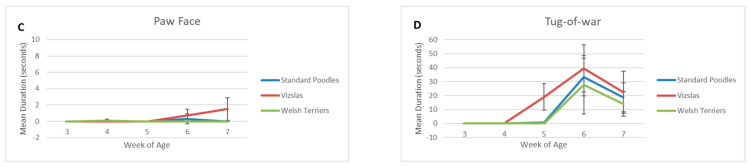
Individual Behaviors in the social (SOC) Play Context. Mean durations in seconds for individual behaviors in the social (SOC) play context for pups across breeds with 95% CI. (**A**) *chase*, (**B**) *keep away*, (**C**) *paw face*, (**D**) *tug-of-war* by age in weeks.

For SOC behavior *chase*, breeds did not differ during weeks 3 (*p* = 1.00), 4 (*p* = 0.67), or 7 (*p* = 0.07). During week 5, Poodles displayed significantly more *chase* than Vizslas (*p* = 0.022) and Terriers (*p* = 0.011) with no difference between Vizslas and Terriers (*p* = 0.70). During week 6, Poodles displayed *chase* significantly more than Vizslas (*p* = 0.008) and Terriers (*p* < 0.001) (Figure 3A). Vizslas were observed to *chase* more than Terriers (*p* < 0.001) and Terriers did not *chase* at all during weeks 3–7 (Figure 3A, Appendix A Table A3).

Breeds did not differ during weeks 3 (*p* = 1.00), 4 (*p* = 0.49) or 5 (*p* = 0.09) in use of *keep away* as none of the breeds exhibited *keep away* during week 3. Poodles at week 4 and Vizslas at week 5 were just starting *keep away* games (Figure 3B, Table A3). During weeks 6 and 7, standard Poodles played more *keep away* than Vizslas (*p* < 0.001, *p* = 0.003) and Terriers (*p* < 0.001, *p* = 0.003). During weeks 6 and 7, there was no difference between Vizslas and Terriers (*p* = 0.10, *p* = 1.00) because Terriers never played *keep away*, whereas Vizslas played *keep away* rarely (Figure 3B, Appendix A Table A3).

Breeds did not differ in use of *paw face* during play weeks 3 (*p* = 1.00), 4 (*p* = 0.62) or 5 (*p* = 0.75), but they did differ during weeks 6 (*p* = 0.040) and 7 (*p* < 0.001). During week 6, Vizslas engaged in *paw face* more than Terriers (*p* = 0.011), while Poodles did not differ from Vizslas (*p* = 0.14) or Terriers (*p* = 0.15). During week 7, Vizslas engaged in *paw face* more than standard Poodles (*p* < 0.001) and Terriers (*p* < 0.001) (Figure 3C, Appendix A Table A4). No differences were found between Poodles and Terriers (*p* = 1.00).

Breeds did not differ in use of *tug-of-war* during play during weeks 3 (*p* = 0.97), 4 (*p* = 0.84), 6 (*p* = 0.15), or 7 (*p* = 0.43). During week 5, Vizslas played more *tug-of-war* than Poodles (*p* = 0.018) and Terriers (*p* = 0.002) (Figure 3D, Appendix A Table A3). No significant differences were found between Poodles and Terriers (*p* = 0.31).

## 4. Discussion

We have herein described in detail an ethogram for studying object play behavior in canids that has been used in previous papers [27,28] on domestic dogs [27] and wolves [28]. This ethogram could potentially provide a framework for evaluating object play in many species, especially the early development of play during the socialization period [43] and the juvenile periods in animals. In this paper, we applied our descriptive systems to seven litters of pups from three breeds of dogs and documented the occurrence of the 31 behavior units from 3–7 weeks of age. We pooled the behavior units into three groups, namely those occurring in solitary (SOL) contexts, social (SOC) contexts, and those occurring in both (BOTH) contexts.

We found the counts and diversity in time spent playing with objects and object-related behaviors increased with age, similar to findings of captive-born wolf pups [28] and other canids [35]. Davis et al. [28] found that the onset of object play in captive wolf pups occurred at 2 weeks of age, one week prior to any of the dog puppies. Biben [35] reported object play onset at 5–7 weeks for captive bush dogs and crab-eating foxes and Pal [48] reported onset of object play in free-ranging dogs at 5 weeks of age. These differences in the onset of object play between the above-mentioned canid species may be related to differences in their environmental pressures, social organizations, and/or hunting strategies. More comparative studies of these and other canids are needed to fully investigate the implications of potential species differences across canids in object play development. Using a comparative ethogram such as ours (Table 1) and the methods laid out in this paper provide a potential methodology for such a broader comparison, as evidenced by our comparison of wolf pups using this ethogram [28].

Across all three breeds in our current study, solitary (SOL) object interactions were the first type of play behavior to appear, and the most common throughout the 3–7-week development period. This supports unpublished data on behavioral development in border collies [39]. There were no differences across breeds in the SOL play context during any of the weeks. Furthermore, all breeds changed across developmental time with the same pattern; an increase in SOL behaviors at weeks 3–4, at the onset of object interaction, and a further increase at weeks 5–6, when social object play emerged. However, there was an overall decrease in SOL play between 6–7 weeks of age as social object play peaked. Compared to wolf pups provided with the same objects during the same 3–7-week period [28], dogs began to exhibit object-directed behavior about one week later and displayed less object-related behavioral diversity, supporting the findings from Lord [45], in which wolves’ sensory systems developed earlier than dogs.

Aspects of predatory behavior can be detected in puppy object play even at only a few weeks old. Although it is tempting to attribute object play to simple exploration and the development of feeding behaviors, such as foraging and hunting, that are so apparent in object play in many birds and mammals [2], the ubiquity of similar behavior in many adult species suggests that rather complex cognitive domains and behavioral systems are involved [2,53]. Indeed, the games dogs play with objects (including with humans [30]) may also be derived from the cooperative hunting behavior of wolf packs. Coppinger [54] suggested solitary play in dogs may be an artifact of predatory sequence disruption created by intense human selection for various non-hunting traits. Livestock-guarding dogs with a more disrupted predatory sequence were found to have less solitary play than retrievers and herders, breeds selected to maintain more of the predatory sequence [16,17,18]. In short, object play may involve a complex mix of motivational systems independent of satisfying specific hunger, social, or other behavioral demands, which appear to be even further modified by human selection pressures for domestic dogs.

The BOTH play context of behavior patterns found in both social and solitary contexts, such as SOL, did not differ significantly by breed within any of the weeks. Across-breed BOTH behaviors had a significant increase between weeks 3–4 at the onset of BOTH behaviors but did not significantly increase or decrease for the weeks 4–5, 5–6, or 6–7. BOTH behaviors did not change in the same way over time as SOL or SOC, and this may be due to a possibly more transitional quality of these behaviors than that of SOL and SOC. Behaviors occurring in BOTH are likely transitional to some social (SOC) only behaviors. For example, *grab-headshake* is a BOTH behavior that develops before SOC only behaviors and is an essential component to *tug-of war*. *Grab-headshake* also changes in form as pups develop and have more motor control; *grab-headshake* starts very uncoordinated with the pup shaking their whole body when they conduct a grab, and it occurs much slower. However, as pups develop more control, they can just shake their head side-to-side while also being able to brace their front feet during a game of *tug-of war*. Hence, some of the BOTH behaviors may truly be ‘transitional’ and necessary for the onset of SOC behaviors. These ‘transitional’ behaviors may also correspond to the onset of more complex play [28] in wolves. SOC play develops later than SOL or BOTH, and this may in part be due to more complex motor, social, and cognitive requirements for this type of play, i.e., combining social behavior and object play together. There are also some SOL behaviors, such as *tear* and *toss*, that emerge and become more common later in development around the time of the emergence of SOC play, and these also require more complex motor skills and possibly cognitive abilities.

The onset of non-object social play toward littermates in dogs and wolves starts with mouthing and wrestling and occurs before the onset of solitary play [28,43] The onset of social interactions involving objects (SOC) lagged behind that of solitary play by 1–2 weeks depending on the breed, a significant effect. This developmental pattern is common in other canid species, such as captive wolves, [28] but not the domestic cat, in which play is initially focused on littermates and the dam, and switches to objects and prey at 6 weeks of age, when all predatory behaviors intensify [55,56]. This phase shift during the onset of object play occurs about 2–3 weeks later in cats than in dogs and wolves, which also coincides with the onset of South American canid species’ solitary object play [35], suggesting the importance of future research. SOC behaviors, such as *chase* and *tug-of-war*, peaked in Poodles and Vizslas at 6 weeks of age. The social behavior of these two breeds significantly decreased at weeks 6–7, while the Terriers’ SOC behaviors increased at week 5–6 but did not change from weeks 6–7. Additional assessment weeks are needed to better determine if social object behaviors peak later for the Terriers.

Within the SOC play context, but not the BOTH or SOL context, additional breed differences began to emerge in individual behaviors *chase*, *keep away*, *tug-of war*, and *paw face*. SOC behaviors’ onset occurs later than behaviors in SOL and BOTH behavior groups, and Terriers were the last to exhibit SOC behaviors. Terriers only played *tug-of war* and very rarely played *chase*. Terriers did not exhibit *keep away* while playing. All breeds exhibited *tug-of war* but did not start this behavior until weeks 5–7. Vizslas exhibited significantly more *tug-of war* than Poodles and Terriers at week 5 as they began this behavior before Poodles and Terriers. By week 6, Poodles caught up to Vizslas in *tug-of war*, and Poodles exhibited *keep away* the most, while *paw face* was the rarest of the SOC behaviors, although it was observed most often in Vizslas. Because the Terriers’ SOC behavior onset and overall amount of play develops later [27], further studies are needed to follow Terriers for a longer time (we have a larger dataset of older Terriers that do exhibit more play and seem to catch up). The behavioral variation seen among the three breeds can be further investigated at older ages and across more dog breeds to see if a pattern emerges.

*Guard* and *tackle*, two other social behaviors listed in our ethogram from the pilot study (which included slightly older and more breeds of pups), were not seen in any of these three breeds during 3–7 weeks, but we did see *guard* in older Poodles [28]. Wolf pups exhibit *guard* aged 3–7 weeks, suggesting differences in the development of play behaviors across taxa that may vary based on natural selection pressures and the effects of domestication [28]. Wolf puppies also seemed generally less interested in object play than social play, and social object play transitioned to play fighting, a pattern also seen in black bear cubs [57]. We did see what is labeled “agonistic” behavior occurring when pups, particularly Poodles, were engaged in social object play, which then transitioned into either social play (e.g., non-agonistic play fighting) or more serious agonistic behavior. The onset of social play and agnostic behavior in several breeds of dogs is reported to develop contemporaneously [38], lending support to these observations and the need for more research to properly classify these emerging behaviors as play fighting, agonistic, or a combination of both. Analysis of these behaviors is beyond the scope of this study, but qualitatively, the behaviors resemble play-fighting or rough-and-tumble play [20]. KMD observed that both dog and wolf pups exhibit “agonistic” behaviors in early development that look more intense, out of context, or have some other motor pattern alteration from the adult representation of the behavior, which could represent play variations (e.g., rough-and-tumble play) or inexperience with appropriate agonistic contexts. Indeed, black bear cubs playing with humans had to learn to restrain their intense scratching and biting during play [57]. Moreover, rough-and-tumble play may help pups learn to modulate their agonistic behaviors so as not to hurt other pups. Puppies isolated from conspecific contact until 12 weeks of age were more likely to show later aggression to other dogs than those reared with littermates [58]. Future studies could look at this switch from social object play to social and agonistic behavior or rough-and-tumble play in pups aged 3–7 weeks.

Several behaviors function as social object play invitations in dogs [39,59,60,61]. *Play bow*, *pounce*, *exaggerated approach* (including *approach-retreat*), and *grab-headshaking* could be behaviors used by one pup to engage another pup in object play. The receiving pup would either reciprocate or reject the solicitation by ignoring or turning or moving away from the inviting pup. Further analysis of the sequential organization of play behavior [53,62] would be a good next step for future studies. These behaviors were placed in the BOTH category as the pups initially displayed the behaviors to the objects, and then to other pups in later weeks with objects incorporated. Social object play endures well into adulthood for dogs and other canid species, and play invitations may serve an additional role of communicating the intention of play between animals, preventing escalation into real aggression [39,58,63].

Play may be considered a window into communication [63]. Most play research has focused on intraspecies social play, but evidence suggests that dogs may use objects to initiate and maintain playful interactions with humans more so than during object play with other dogs [64]. Dogs have evolved to have a heightened interspecific (human–dog) responsiveness, though this is not necessarily a sign of cognitive advancement [65]; rather, this may be a shift through artificial selection from conspecific cooperation in the ancestor (the wolf) to human–dog cooperation [66]. Regardless, dogs are highly skilled at perceiving a range of human communication signals and the ability to understand human gestures is apparent even in young puppies with little human experience [67].

The degree of adult cooperative hunting can be detected in pup object play for a given canid species [35]. Young bush dogs, who cooperatively hunt large prey as adults, demonstrated more peaceful group object play, whereas object play in pups of the less-social opportunistic hunting crab-eating fox tended to be more agonistic. Given the wide behavioral diversity in dogs, it is not surprising that we found some early differences amongst the breeds in their social object play behaviors. Poodles and Vizslas, for example, both originated as retrieving dogs, but for many decades, the standard Poodle has been one of the most popular companion-only dogs, whereas the Vizsla continues to be bred for hunting. Behaviors such as *keep-away* and *chase*, which are ostensibly more socially engaging than behaviors such as *paw*, were more common in the Poodle during several weeks of our study. Conspecific cooperation in the wolf ancestor may have shifted to human–dog cooperation in dogs [66], and the degree of adult cooperative hunting can be detected in pup object play in wild canids [35]. Therefore, it may be a useful for future studies of object play development to investigate the development of human–dog object play, as well as dog–dog object play across more companion breeds, with comparisons to wild canid development where possible. Additionally, future studies with additional litters and companion breeds may find a stronger correlation between certain social object behaviors in young pups and strong affiliative behavior to humans. Moreover, other social and even solitary behaviors observed early in development could likely be associated with strong herding, guarding, companion, or possibly service performance in adult dogs.

Object curiosity and manipulation seems to precede play [12], and we also found this sequence in puppies. Object play rapidly takes on more complexity and, in many species, begins to closely resemble adult behaviors within the first few weeks of life [23]. In domestic cats, object play primarily consists of predatory behavior patterns. Its onset follows social play and coincides with weaning in kittens [67,68]. Stone handling, which can be observed in several macaque species, is complex, but all elements can be seen in foraging behavior (nut cracking) [53,69]. Object play is often more common in juvenile members of the species, prompting early researchers to posit that play is an immature version of adult behavior. However, most adults of these species also perform alternative versions of the behavior that fit the five criteria for play [2], prompting newer theoretical frameworks focused on the incorporation of existing functional behaviors into a “play behavioral system” that may have connected or completely separate motivations from their behavioral counterparts in foraging or courtship [53].

### Limitations

Although our ethogram was extensive and our continuous sampling method yielded detailed study of pup development, a limitation was the lack of vocalization data. Our technology did not allow us to determine which pup in a group was vocalizing. Additionally, the small number of litters enrolled in our study hindered our ability to detect any significant breed or age effects for individual behaviors within SOL or BOTH (transitional) play contexts. Some behavior categories, particularly our SOL play context category, included a large number of individual behaviors with varying rates of change, which may have obscured any differences. Future studies should focus on individual behaviors in the SOL and BOTH groups in greater detail.

The limited number of breeds we recruited for this study restricted our ability to generalize findings to other breeds within a functional category (e.g., herding, guarding, companion). Litter sizes varied within breed and therefore could be a confounding variable. We have collected data on additional breeds that could alleviate these issues in future studies, not only by providing more information on more diverse breeds, but also by increasing power for a more detailed analysis of individual behavior and possibly sex differences in object play. Although we controlled for early environmental effects by collecting data from litters that were raised in similar levels of enriched environments, there were inevitability some environmental differences. This study was designed to provide a method and ethogram for continued comparative work and it was beyond the scope of this study to look at how environmental factors during early ontogeny influence the development of play.

## 5. Conclusions

This study provides a detailed ethogram for use in ontogenetic studies of object play in canids. We compared the development of object play in Vizslas, standard Poodles, and Welsh Terrier domestic dogs from 3–7 weeks of age and grouped the individual behaviors into three play contexts. Solitary/SOL behaviors developed first for all breeds and increased in diversity with age. BOTH behaviors appeared to be transitional to behavior in the social/SOC group (social object play). The SOL and BOTH behaviors did change significantly with developmental time but followed the same pattern for all breeds. SOC behaviors developed last, and the individual SOC behaviors exhibited differed significantly among breeds. Lastly, Welsh Terriers had a phase shift to later development of play compared to Vizslas and Poodles. Ontogenetic differences in the type and frequency of object play behaviors across breeds may aid in understanding both how play develops and the role that selection and domestication has played in the evolution of dogs and their behavioral variability.

## Figures and Tables

**Table 3:** Total Frequency of Individual Behaviors. Total counts and durations (in seconds) are listed for each behavior by breed collapsed across total observation periods (3–7 weeks).

		Breed			
		Welsh Terriers	Standard Poodles	Vizslas	Total
		N = 11	N = 17	N = 14	N = 42
Agonistic	Count	0	0	3	3
	Duration	0.0	0.0	10.5	10.5
Approach	Count	52	122	118	292
	Duration	73.3	181.1	114.9	369.3
Approach-retreat	Count	7	6	10	23
	Duration	30.5	6.7	23.5	60.6
Avoid	Count	0	1	0	1
	Duration	0.0	3.6	0.0	3.6
Bite	Count	168	213	161	542
	Duration	491.2	651.8	454.4	1597.5
Carry	Count	19	145	138	302
	Duration	47.6	512.2	567.3	1127.2
Chase	Count	11	97	28	136
	Duration	32.0	316.4	89.3	437.7
Chew	Count	850	1049	1168	3067
	Duration	7819.1	9465.1	15,338.3	32,622.5
Dig	Count	5	2	0	7
	Duration	20.2	6.0	0.0	26.2
Drag	Count	73	117	46	236
	Duration	271.0	524.0	135.8	930.8
Exaggerated Approach	Count	10	28	8	46
	Duration	6.0	67.0	14.0	87.0
Grab	Count	127	257	190	574
	Duration	316.0	626.7	565.9	1508.5
Grab-headshake	Count	127	118	197	442
	Duration	340.5	293.5	435.8	1069.8
Hold Object	Count	97	146	176	419
	Duration	461.4	519.3	709.8	1690.4
Keep Away	Count	0	58	12	70
	Duration	0.0	221.2	43.1	264.3
Leap	Count	1	7	3	11
	Duration	0.3	3.7	2.6	6.6
Lick	Count	71	55	26	152
	Duration	391.9	238.7	116.0	746.6
Lie on Object	Count	10	0	0	10
	Duration	53.7	0.0	0.0	53.7
Nose	Count	896	954	1202	3052
	Duration	3080.0	3725.2	4328.1	11,133.2
Paw	Count	182	262	188	632
	Duration	477.4	777.2	425.2	1679.7
Paw Face	Count	1	2	10	13
	Duration	2.2	9.5	44.1	55.8
Pickup and Drop	Count	2	28	15	45
	Duration	5.9	104.5	34.5	144.8
Play Bow	Count	10	10	16	36
	Duration	15.3	24.6	22.2	62.1
Pounce	Count	14	18	13	45
	Duration	11.8	99.4	14.1	125.3
Stand Over	Count	12	0	0	12
	Duration	47.1	0.0	0.0	47.1
Tackle	Count	0	2	0	2
	Duration	0.0	4.6	0.0	4.6
Tear	Count	69	35	78	182
	Duration	553.0	298.7	734.5	1586.2
Toss	Count	0	1	0	1
	Duration	0.0	2.0	0.0	2.0
Tug-of-war	Count	74	204	218	496
	Duration	763.7	1473.6	1891.1	4128.3
Tug-pull	Count	43	66	46	155
	Duration	169.1	371.4	202.2	742.6

**Table 4:** Number of pups performing behaviors by age. Number of pups (out of 42 total) that performed each behavior at least once at each week (7 litters and 3 breeds combined) from weeks 3–7. Note that at week 7, the total number of pups was 41 due to one pup being rehomed that week.

	Age in Weeks (Pup N = 42 Weeks 3–6.5 and N = 41 Week 7)								
Behaviour	3.0	3.5	4.0	4.5	5.0	5.5	6.0	6.5	7.0
Agonistic	1	0	0	0	2	0	0	0	0
Approach	8	10	7	16	14	16	19	30	24
Approach-retreat	1	1	0	2	3	0	1	5	2
Avoid	0	0	0	0	0	0	0	1	0
Bite	7	5	15	19	31	27	24	34	30
Carry	0	0	2	11	15	12	22	25	18
Chase	0	0	1	2	7	3	21	22	15
Chew	9	13	24	34	42	42	42	42	41
Digging	0	0	1	0	1	1	0	1	2
Drag	1	0	2	13	21	12	26	22	19
Exaggerated Approach	0	2	0	1	10	4	6	3	9
Grab	1	2	8	17	27	20	30	40	29
Grab-headshake	0	1	9	20	25	15	30	42	29
Hold Object	0	4	3	13	27	17	30	38	29
Keep Away	0	0	0	4	3	3	11	6	6
Leap	0	0	0	0	1	0	4	1	2
Lick	5	8	13	11	7	8	8	11	12
Lie on Object	0	3	3	5	5	0	6	6	5
Nose	40	42	42	42	42	42	42	42	41
Paw	3	17	26	42	42	42	38	42	39
Paw Face	0	0	0	1	2	0	1	4	5
Pickup and Drop	1	1	0	2	5	3	2	9	7
Play Bow	0	3	3	3	8	4	3	3	2
Pounce	3	0	1	1	7	4	7	7	4
Stand Over	0	3	6	11	7	5	2	4	3
Tackle	0	0	1	0	0	0	1	0	0
Tear	0	0	3	10	13	8	11	14	12
Toss	0	0	0	0	0	0	0	1	0
Tug-of-war	0	0	0	3	6	14	35	32	28
Tug-pull	3	0	0	3	10	12	26	24	16

**Table 5:** Play Context Means. Means and standard deviations for play context: solitary only (SOL); social only (SOC); and behaviors that occur in both contexts (B) for each breed (standard Poodles, Vizslas, and Welsh Terriers) from 3–7 weeks.

Play Context	Age (in Weeks)	Standard Poodles		Vizslas		Welsh Terriers	
		Mean	Standard Deviation	Mean	Standard Deviation	Mean	Standard Deviation
Solitary	3.0	27.39	27.47	43.92	30.71	34.93	26.87
	4.0	90.32	72	135.45	96.97	168.92	49.58
	5.0	159.72	69.05	245.81	178.7	120.96	54.12
	6.0	165.74	61.24	263.01	75.46	219.22	64.32
	7.0	148.64	93.08	247.72	97.24	151.01	108.35
Both	3.0	3.36	7.12	3.4	3.71	6.6	14.05
	4.0	7.16	7.67	6.25	7.84	8.68	5.89
	5.0	11.89	12.33	9.52	7.37	11	9.33
	6.0	13.02	6.96	9.37	8.32	9.57	9.06
	7.0	10.62	8.53	11.41	10.51	14.02	10.15
Social	3.0	0	0	0.03	0.1	0	0
	4.0	0.96	1.52	1.04	2.72	0.15	0.51
	5.0	2.94	3.15	19.29	17.42	0	0
	6.0	43.64	30	42.84	32.4	28.1	35.93
	7.0	29.21	27.08	24.96	31.03	16.79	15.96

## Data Availability

The data presented in this study are available in the text and as Appendix A Table A1, Table A2, Table A3, Table A4 and Table A5.

## Appendix A

**Table A1:** Pairwise average Cohen’s kappa for observer pairs. The first column includes the average kappa for each observer pair across all breed observations for the behaviors exhibited, sequence of behaviors, and number of bouts calculated as a combined kappa (labeled ‘Behaviors’). Kappa for duration and duration sequence are also reported for each observer pair. Cohen’s kappa averaged across all observer pairs for each overall kappa (behaviors, duration, and duration sequence) are noted at the bottom of the column.

Observer Pairs	Behaviors	Duration	Duration Sequence
GSS/AMS	0.75	0.94	0.96
CW/GSS	0.82	0.96	0.97
AMS/TA	0.83	0.94	0.96
TA/TV	0.76	0.94	0.96
TA/GSS	0.72	0.95	0.96
CW/TV	0.83	0.92	0.95
TV/GSS	0.74	0.95	0.97
AMS/TV	0.91	0.96	0.96
CW/TA	0.83	0.95	0.97
**Overall Kappa**	**0.80**	**0.94**	**0.96**

**Table A2:** Individual behaviors in the SOL play context for each breed. Mean and standard deviation of duration data for individual behaviors in the (solitary/SOL) play context for each breed at 3–7 weeks of age.

SOL Behaviors	Age (in Weeks)	Standard Poodles		Vizslas		Welsh Terriers	
		Mean	Standard Deviation	Mean	Standard Deviation	Mean	Standard Deviation
Pickup and Drop	3	0	0	0.2	0.7	0	0
	4	0.4	1.3	0	0	0	0
	5	0.7	1.2	0	0	0	0
	6	1.8	4.1	0.6	1.1	0.2	0.6
	7	0.2	0.6	0.7	1.3	0.1	0.5
Bite	3	0	0	0.8	0.9	2.5	4.8
	4	5.1	5	0.8	1.5	5	6
	5	5.3	4.1	4.2	6.2	2.8	2.4
	6	6.3	5.8	9.7	10.8	5	4.9
	7	5	7.1	1.6	3	14.2	22.5
Carry	3	0	0	0	0	0	0
	4	0.9	2.8	3	4.5	0.2	0.5
	5	2.7	4.2	7.6	8.8	0.5	0.9
	6	7.9	7.2	5.6	6	0.8	0.9
	7	6.9	10.7	7.2	15	1.2	2.2
Chew	3	4.9	7.1	5.3	6.2	4.5	8.6
	4	48.1	59.8	63.2	57.7	71.1	57.6
	5	84.5	52.4	176.2	144.4	70.5	43.8
	6	92.8	55.1	182.4	91.7	163.6	59.3
	7	96.1	85.6	202.4	87.4	91.5	102
Drag	3	0	0.2	0	0	0	0
	4	2.2	4.1	0.5	1.1	4	8.3
	5	4.8	6.1	1	2.5	2.5	3.5
	6	7.9	6	1.9	3.6	3.5	3.9
	7	0.9	1.7	2.4	3.1	4.6	6.9
Grab	3	0	0	0.2	0.9	0.5	1.5
	4	1	1.9	2.4	3.6	3.7	5.3
	5	4.2	4.9	4.2	5.3	3.1	2.4
	6	12	7.4	12.6	9	3.9	2.7
	7	2.4	3.5	4	4	6.5	7.1
Hold Object	3	0	0	0.2	0.7	0.9	2.6
	4	0.7	1.3	2	4.4	5.4	9.2
	5	6.4	6.6	2	6.1	6.4	4.9
	6	7.3	8.7	16.5	16.1	5.4	11
	7	1.6	3.5	8.4	11.1	5.7	6.5
Lick	3	2.1	4.4	2	5.4	6.9	12.6
	4	2	1.7	0.8	2	4.5	6.8
	5	1.7	2.8	0	0	4.8	7.7
	6	1	1.8	0.8	1.1	1.2	1.9
	7	0.4	1.1	1.2	1.9	0.8	2.2
Nose	3	20.3	23.6	34.9	23.6	19.7	10.4
	4	27.6	18.1	61.2	49	67.7	21.1
	5	39.7	60.5	32	35.9	21.1	19.6
	6	19.3	10.4	17.3	8.7	23.2	9.7
	7	5.2	5.1	10.3	7.5	16.5	12.7
Pounce	3	0	0	0.2	0.3	0	0
	4	0	0	0	0	0.1	0.3
	5	0.5	1.6	0.3	0.6	0.1	0.2
	6	2.5	8.2	0.1	0.2	0	0.1
	7	0.1	0.5	0.1	0.3	0	0
Tear	3	0	0	0	0	0	0
	4	1.9	4.7	1.2	2.5	6.7	8.8
	5	3.8	8.7	18.3	45.1	8.4	10.1
	6	2.2	3.7	11.8	13.5	7.4	17.4
	7	1.6	4.7	4.9	8.7	5.4	15.2
Tug-pull	3	0	0	0	0	0	0
	4	0.1	0.6	0.5	1.7	0.2	0.7
	5	5.4	6.9	0	0	0.1	0.4
	6	4.5	4.4	3.9	5.4	3.2	6.6
	7	1.9	2.7	4.5	7.4	2.2	4

**Table A3:** Individual behaviors in the SOC play context for each breed. Mean and standard deviation of duration data for individual behaviors in the (social/SOC) group at 3–7 weeks of age.

SOC Behaviors	Age (in Weeks)	Standard Poodles		Vizslas		Welsh Terriers	
		Mean	Standard Deviation	Mean	Standard Deviation	Mean	Standard Deviation
Chase	3	0	0	0	0	0	0
	4	0.3	1	0.7	2.7	0	0
	5	1	2	0	0.2	0	0
	6	6.9	5.6	1.7	2.1	0.2	0.5
	7	2.1	3	1.1	3.3	2.6	4
Keep Away	3	0	0	0	0	0	0
	4	0.4	1.1	0.2	0.8	0	0
	5	1.2	2.4	0	0	0	0
	6	3.2	2.9	1	2	0	0
	7	3.4	6.5	0	0	0	0
Leap	3	0	0	0	0	0	0
	4	0	0	0	0	1.2	0.4
	5	0	0	0	0	0	0
	6	0	0	0	0	1.9	1.0
	7	0	0	0	0	0.9	0.4
Paw Face	3	0	0	0	0	0	0
	4	0	0	0	0	0.1	0.3
	5	0	0	0	0.1	0	0
	6	0.3	1.1	0.7	1.6	0	0
	7	0	0	1.5	2.6	0	0
Tug-of-war	3	0	0	0	0	0	0
	4	0.2	0.7	0.1	0.4	0	0
	5	0.7	1.4	18.9	17.8	0	0
	6	33.2	28.4	39.4	32.3	27.7	35.5
	7	18.6	21.9	22.4	28.8	14	15

**Table A4:** Individual behaviors in the BOTH play context for each breed. Mean and standard deviation of duration data for individual behaviors in the (both/BOTH) group at 3–7 weeks of age.

BOTH Behaviors	Age (in Weeks)	Standard Poodles		Vizslas		Welsh Terriers	
		Mean	Standard Deviation	Mean	Standard Deviation	Mean	Standard Deviation
Approach	3	2.4	6.7	1.1	1.6	0.3	1
	4	0.2	0.4	1	2.7	1	2
	5	1.3	1.6	0.9	1.2	0.2	0.3
	6	0.9	0.6	1.1	1.2	0.4	1.1
	7	0.9	1.7	0.4	0.5	2.9	5.1
Approach-exaggerated	3	0	0	0.3	0.7	0.7	2.2
	4	0.2	0.5	0	0	0	0
	5	0.9	2.2	0.1	0.2	0.8	2.2
	6	0.7	0.9	0.9	3.4	0	0.1
	7	0.9	2.2	0	0	0.4	1.3
Grab-headshake	3	0	0	0.2	0.8	0	0
	4	1.2	2.6	3.7	7.3	2.5	4.3
	5	1.2	1.9	4.3	5.9	4.3	4
	6	4.1	5	4.2	3.2	5.5	5.3
	7	4.2	6.1	3.5	5.3	6.3	7
Paw	3	1	2.5	1.6	2	5.6	14.3
	4	5.5	5.5	1.4	2	4.7	4.2
	5	8.3	10.4	3.8	2.8	5.5	5.6
	6	6.8	4.7	3.1	4.6	3.7	5.6
	7	2.4	4.7	7.5	8.2	4.4	7.3
Play bow	3	0	0	0.2	0.3	0	0
	4	0.1	0.4	0.1	0.4	0.5	1.6
	5	0.2	0.5	0.5	1.6	0.2	0.4
	6	0.3	0.8	0.1	0.3	0	0
	7	0.2	1	0.1	0.2	0	0

**Table A5:** Bi-weekly Frequency of Individual behaviors collapsed across breed. Total counts and duration (in seconds) for each behavior at each week and mid-week time point collapsed across breed. Total number of pups across breeds was 42 (7 litters and 3 breeds combined) from weeks 3–6.5. Note that at week 7, one pup had been rehomed so the number of pups is 41 on week 7.

Behavior		Age in Weeks (Pup N = 42 Weeks 3–6.5 and N = 41 Week 7)								
		3.0	3.5	4.0	4.5	5.0	5.5	6.0	6.5	7.0
Agonistic	Count	1	0	0	0	2	0	0	0	0
	Duration	0.8	0.0	0.0	0.0	9.7	0.0	0.0	0.0	0.0
Approach	Count	23	26	8	25	33	29	32	66	50
	Duration	25.5	93.2	5.1	52.7	20.4	49.4	24.0	46.0	53.0
Approach-retreat	Count	1	2	0	2	5	0	1	10	2
	Duration	0.3	14.4	0.0	2.1	15.4	0.0	2.0	22.9	3.5
Avoid	Count	0	0	0	0	0	0	0	1	0
	Duration	0.0	0.0	0.0	0.0	0.0	0.0	0.0	3.6	0.0
Bite	Count	11	11	35	55	77	66	74	125	88
	Duration	18.5	58.6	134.2	168.7	166.9	189.2	222.6	374.8	264.1
Carry	Count	0	0	2	19	45	37	59	80	60
	Duration	0.0	0.0	11.1	109.8	192.4	137.8	195.6	248.6	231.9
Chase	Count	0	0	1	3	8	4	59	34	27
	Duration	0.0	0.0	7.9	22.7	26.9	15.4	193.1	92.9	78.8
Chew	Count	21	49	119	303	444	457	560	584	530
	Duration	149.8	265.3	1548.5	3423.1	4506.5	5393.2	6371.0	5491.9	5473.2
Dig	Count	0	0	2	0	1	1	0	1	2
	Duration	0.0	0.0	6.0	0.0	7.8	2.1	0.0	2.2	8.0
Drag	Count	1	0	4	26	50	19	61	43	32
	Duration	1.3	0.0	22.2	154.0	171.6	81.6	269.1	132.3	98.7
Exaggerated Approach	Count	0	3	0	2	12	4	8	5	12
	Duration	0.0	7.8	0.0	3.0	27.2	7.2	17.4	8.1	16.3
Grab	Count	1	3	15	39	73	46	118	196	83
	Duration	1.0	15.8	50.3	131.9	160.5	136.3	346.4	497.8	168.5
Grab-headshake	Count	0	4	17	46	89	53	67	95	71
	Duration	0.0	5.7	42.3	158.5	186.1	108.2	176.9	201.2	190.9
Hold Object	Count	0	6	3	37	51	50	97	114	61
	Duration	0.0	26.7	9.6	189.7	216.4	210.7	383.2	446.5	207.6
Keep Away	Count	0	0	0	5	3	7	27	10	18
	Duration	0.0	0.0	0.0	21.0	18.0	31.8	106.4	29.5	57.6
Leap	Count	0	0	0	0	1	0	7	1	2
	Duration	0.0	0.0	0.0	0.0	1.0	0.0	3.7	0.3	1.5
Lick	Count	11	24	16	31	17	14	13	12	14
	Duration	103.2	177.4	47.1	140.5	91.2	72.1	43.3	38.9	32.9
Lie on Object	Count	0	1	0	7	1	0	0	0	1
	Duration	0.0	6.8	0.0	33.4	8.5	0.0	0.0	0.0	5.0
Nose	Count	275	231	507	460	418	320	300	336	205
	Duration	1278.6	823.4	2419.0	1721.5	1254.3	1569.5	905.1	748.2	413.5
Paw	Count	4	37	36	86	109	102	87	104	67
	Duration	15.7	187.2	94.7	233.2	229.9	325.3	211.8	187.9	194.0
Paw Face	Count	0	0	0	1	2	0	1	4	5
	Duration	0.0	0.0	0.0	2.2	1.8	0.0	2.6	27.6	21.5
Pickup and Drop	Count	1	1	0	4	7	4	2	18	8
	Duration	2.6	3.0	0.0	15.1	19.8	7.5	6.5	75.9	14.4
Play Bow	Count	0	4	3	5	9	7	3	3	2
	Duration	0.0	4.7	6.5	11.4	9.6	11.0	11.0	2.8	5.0
Pounce	Count	3	0	2	1	11	4	11	7	6
	Duration	4.4	0.0	1.2	1.7	20.4	5.1	80.9	7.9	3.7
Stand Over	Count	0	2	0	3	7	0	0	0	0
	Duration	0.0	2.2	0.0	20.2	24.7	0.0	0.0	0.0	0.0
Tackle	Count	0	0	1	0	0	0	1	0	0
	Duration	0.0	0.0	1.8	0.0	0.0	0.0	2.8	0.0	0.0
Tear	Count	0	0	6	24	35	29	18	47	23
	Duration	0.0	0.0	44.5	201.1	273.8	342.9	173.7	394.5	155.8
Toss	Count	0	0	0	0	0	0	0	1	0
	Duration	0.0	0.0	0.0	0.0	0.0	0.0	0.0	2.0	0.0
Tug-of-war	Count	0	0	0	3	18	43	167	167	98
	Duration	0.0	0.0	0.0	8.5	158.3	337.2	1482.6	1358.3	783.4
Tug-pull	Count	0	0	0	6	16	22	44	36	31
	Duration	0.0	0.0	0.0	30.4	57.7	152.0	227.8	136.7	138.0
